# Metastatic pattern of ovarian cancer delineated by tracing the evolution of mitochondrial DNA mutations

**DOI:** 10.1038/s12276-023-01011-2

**Published:** 2023-07-03

**Authors:** Zhiyang Xu, Kaixiang Zhou, Zhenni Wang, Yang Liu, Xingguo Wang, Tian Gao, Fanfan Xie, Qing Yuan, Xiwen Gu, Shujuan Liu, Jinliang Xing

**Affiliations:** 1 Department of Obstetrics and Gynecology, Xijing Hospital, Fourth Military Medical University, Xi’an, China; 2 State Key Laboratory of Cancer Biology and Department of Physiology and Pathophysiology, Fourth Military Medical University, Xi’an, China; 3 State Key Laboratory of Cancer Biology and Department of Pathology, Xijing Hospital and School of Basic Medicine, Fourth Military Medical University, Xi’an, China

**Keywords:** Ovarian cancer, Cancer genetics

## Abstract

Ovarian cancer (OC) is the most lethal gynecologic tumor and is characterized by a high rate of metastasis. Challenges in accurately delineating the metastatic pattern have greatly restricted the improvement of treatment in OC patients. An increasing number of studies have leveraged mitochondrial DNA (mtDNA) mutations as efficient lineage-tracing markers of tumor clonality. We applied multiregional sampling and high-depth mtDNA sequencing to determine the metastatic patterns in advanced-stage OC patients. Somatic mtDNA mutations were profiled from a total of 195 primary and 200 metastatic tumor tissue samples from 35 OC patients. Our results revealed remarkable sample-level and patient-level heterogeneity. In addition, distinct mtDNA mutational patterns were observed between primary and metastatic OC tissues. Further analysis identified the different mutational spectra between shared and private mutations among primary and metastatic OC tissues. Analysis of the clonality index calculated based on mtDNA mutations supported a monoclonal tumor origin in 14 of 16 patients with bilateral ovarian cancers. Notably, mtDNA-based spatial phylogenetic analysis revealed distinct patterns of OC metastasis, in which a linear metastatic pattern exhibited a low degree of mtDNA mutation heterogeneity and a short evolutionary distance, whereas a parallel metastatic pattern showed the opposite trend. Moreover, a mtDNA-based tumor evolutionary score (MTEs) related to different metastatic patterns was defined. Our data showed that patients with different MTESs responded differently to combined debulking surgery and chemotherapy. Finally, we observed that tumor-derived mtDNA mutations were more likely to be detected in ascitic fluid than in plasma samples. Our study presents an explicit view of the OC metastatic pattern, which sheds light on efficient treatment for OC patients.

## Ovarian cancer: Mitochondrial mutations reveal cancer progression

Analysis of mitochondrial DNA offers new insights into cancer spread (metastasis) that could lead to more accurate prognoses and inform selection of better treatment regimens. Mitochondria carry their own DNA, which accumulates mutations at a far higher rate than does chromosomal DNA. These mutations can be extremely useful for tracing cellular lineages. Researchers led by Jinliang Xing and Shujuan Liu at the Fourth Military Medical University in Xi’an, China, have shown that these changes can also provide insights on tumor progression. They analyzed samples from 35 patients with ovarian cancer, revealing distinct differences between the mutational profiles of primary ovarian tumors and metastatic growths. Metastases that differed especially strongly from primary tissue responded more poorly to treatment. Future mitochondrial analyses could yield a better understanding of metastatic spread and the treatment of aggressive cancers.

## Introduction

Ovarian cancer (OC) is a leading cause of death among gynecological malignant tumors globally^1^. Owing to the lack of distinct physical barriers in the peritoneal cavity, OC typically displays early and widespread metastasis at distal intraperitoneal sites, resulting in notable morbidity and mortality. OC metastasis is considered an evolutionary process through which tumor cells acquire a metastatic phenotype via a series of genetic alterations^2^. Accurate reconstruction of the evolutionary trajectory during OC metastatic dissemination holds great promise for revealing novel therapeutic vulnerabilities and providing novel treatment strategies^3^. With the advent of next-generation sequencing (NGS), the landscape and potential therapeutic targets in primary OC patients have been systematically identified^4^. However, far fewer multiregion sequencing studies have been performed to compare the genetic differences between primary and metastatic OC tumors. Recently, based on multiregion whole-exome sequencing, Masoodi et al. investigated the evolutionary history of OC metastasis in six OC patients by analyzing copy number variations (CNVs) and single-nucleotide variants (SNVs)^5^. However, all current studies are limited to investigating only the nuclear genome in a small patient cohort, partially owing to the relatively high cost and complex sequencing data, which greatly reduces the credibility in delineating the OC metastatic pattern and further impedes its large-scale clinical application.

Circular mitochondrial DNA (mtDNA) encodes 2 rRNAs, 22 tRNAs, and 13 proteins essential for oxidative phosphorylation^6^. Due to the lack of histone protection and an inefficient damage repair system, mtDNA has a higher mutation rate than nuclear DNA (nDNA), rendering the detection of mtDNA variations more cost-effective and convenient^7^. The involvement of mtDNA in tumor evolution has long been suspected since altered energy metabolism is a prominent signature of cancer^8^. Critically, increasing studies have leveraged mtDNA mutations as endogenous markers to discriminate different subclonal populations^9,10^, which provides an unbiased approach to investigate tumor clonal evolution. For instance, Kazdal et al. comprehensively revealed the subclonal evolution of pulmonary adenocarcinomas by analyzing the spatial distribution of somatic mtDNA mutations^11^.

Based on the growing evidence supporting the versatile role of mtDNA mutations in clone tracking, the systematic elaboration of OC metastatic patterns based on multiregion mtDNA mutation profiling was carried out in the present study. In addition, the clonal origins of bilateral ovarian tumors were clarified, and the clinical relevance between different metastatic patterns and treatment efficacy was explored. Our study might further guide precise treatment strategies for OC patients.

## Methods

### Patient enrollment and multiregional sampling

Between January 2021 and February 2022, a total of 35 OC patients were recruited from Xijing Hospital, Fourth Military Medical University in Xi’an, China. Patient enrollment criteria were as follows: (1) histopathologically diagnosed with high-grade serous ovarian cancer (HGSOC); (2) FIGO stage III or IV; (3) no prior treatment before sampling; (4) undergoing debulking surgery and six courses of adjuvant chemotherapy; and (5) no history of other malignancies. The clinical characteristics and sampling information are summarized in Table 1. For each patient, primary tumor tissues, paired multiregion metastatic tumor tissues and preoperative blood samples were collected. Adjacent nontumor tissue samples from six patients and ascites samples from five patients were accessible and collected. The abbreviations of different sampling regions are listed in Supplementary Table 1.

**Table 1:** Clinical characteristics of OC patients.

Patients	Age	Histopathology	FIGO stage	Total courses of chemotherapy	Sampling number	Sampling sites
Patient 1	65	HGSC	3	8	7	pROV(1-6) mOM4
Patient 2	56	HGSC	3	6	6	pLOV(1-5) mOM(1-3) ROV(1,2,4),CP
Patient 3	59	HGSC	4	6	16	pLOV(1-2) pROV mDOU mHRR mLPS mOM(1-8) mPER mREC
Patient 4	48	HGSC	4	6	5	pLOV pROV pROV2 mLIV mOM(1-2)
Patient 5	51	HGSC	4	6	9	pLOV pROV mHRR mLIC mLIL mOM mPER mRPS mSIG
Patient 6	65	HGSC	4	6	21	pLOV pROV pROV2 mDOU mLPR mOM(0-11) mRDI mSIG mSIN mVPR
Patient 7	50	HGSC	3	6	7	pLOV pLOV2 pROV(1-3) mOM mTRC
Patient 8	47	HGSC	4	6	11	pLOV(1,3) pROV pROV3 mILE mLIV mOM mPS mRCO(1-2) mVPR
Patient 9	55	HGSC	4	6	10	pOV pOV2 mDIA mLIP mOM mPAN mRCO mSIG(1-2) mSPH mSPL
Patient 10	44	HGSC	4	6	9	pLOV pLOV2 pROV pROV2 mILE mLIV mOM mRCO mSIG mSIN mSPH
Patient 11	39	HGSC	3	6	12	pLOV(1-2) pROV(1-2) mAPD mCOP mDOU mLFT(1-2) mOM mPA mRPR mSIGS mVPR
Patient 12	47	HGSC	3	6	5	pLFT pLFT4 mREC mSIG mVPR
Patient 13	52	HGSC	3	6	5	pLOV pLOV2 pROV pROV2 mLIG mMES mSIG(1-2) mOM2
Patient 14	57	HGSC	4	6	11	pLOV pROV(1-3) mAPD mDOU mLOM mLTH(1-2) mMES mOM mRPS mSPH mTRC
Patient 15	58	HGSC	4	6	12	pLOV(1-2) pROV(1-4) mLPA mLPS mOM(1-3) mPA mRDI mREC
Patient 16	54	HGSC	4	6	11	pLOV(1,2,5,6) pROV(1,2,4,5) mOM(1-3)
Patient 17	41	HGSC	3	8	21	pLOV(1-5) pROV(1-5) mGAF mILE mOM mOM(1-4) mSIG(1-4) mSPH
Patient 18	60	HGSC	3	6	8	pLOV(1-2) pRFT(1,3,4) pROV(2,3,6)
Patient 19	59	HGSC	3	6	9	pLOV(1-2) pROV(1-4) mOM(1-3)
Patient 20	56	HGSC	4	6	6	pLOV(1-2) pROV(1-4)
Patient 21	64	HGSC	3	6	12	pLOV(1-4) pROV(1-3) mOM(1-5)
Patient 22	55	HGSC	4	/	5	pLOV(1-3) pROV(1-4) mPER
Patient 23	50	HGSC	3	6	10	pLOV(1-2) pROV(1-2) mMES mOM(1-2) mREC(1-2) mRPS
Patient 24	49	HGSC	3	6	8	pLOV(1-2,4) pROV(1-4) mOM(1-3) CP
Patient 25	65	HGSC	3	6	6	pOV(1-7) mDOU mHRR mLPS
Patient 26	79	HGSC	3	6	10	pLOV(1-3) pROV mGAB mMES mREC mLPA CP
Patient 27	64	HGSC	4	6	24	pLOV(1-12) mDOU mLTH mMES mOM(2-3) mROV mSIG mSPH mSPR mVPR
Patient 28	56	HGSC	3	6	16	pLOV(1-7) mOM(1-3) pROV(1-4,6) CP
Patient 29	54	HGSC	3	6	21	pLOV(1-7,9-12) mDOU mMES mSIG mSIP(1-3) pROV(1-4)
Patient 30	52	HGSC	3	6	5	mLPS mOM(1-2)mSIG mSIN
Patient 31	50	HGSC	3	8	21	pLOV(1,2.4.7) mOM2 pROV1
Patient 32	71	HGSC	3	6	9	pROV(1-2) mLEL mLIG mOM mPER mRPA CP
Patient 33	59	HGSC	3	6	9	pLOV mRDI pROV(1-5) mRRS CP
Patient 34	44	HGSC	3	6	16	pROV(1,2,4-6) mDOU(1-2) mHRR mLIP(1,3) mMES(1-2) mRVL mVPR
Patient 35	71	HGSC	4	12	25	mLN(1-3,5,10) mROC mRPR mSIG pROV(2-7,9-12)

### Sample processing and DNA extraction

The hematoxylin and eosin (H&E) slides of each selected tissue sample for DNA extraction and sequencing were carefully reviewed by two independent pathologists to ensure that the tumor cell content was at least 80% and that there were no tumor cells in the adjacent nontumor samples. For tissue samples with an insufficient percentage of tumor cells, macrodissection was carried out on frozen sections based on H&E staining to ensure a tumor cell percentage of at least 80%. Blood and ascites samples were subjected to two steps of centrifugation to separate the supernatant from the precipitate^12,13^. Genomic DNA was extracted from multiple region samplings of tumor tissue, adjacent nontumor tissue and peripheral blood mononuclear cell (PBMC) samples using an EZNA DNA kit (Omega, USA). Cell-free DNA (cf-DNA) was extracted from plasma and supernatant samples of ascites using a QIAamp circulating nucleic acid kit (QIAGEN, USA). In addition, DNA was extracted from ascites precipitate using a QIAamp mini kit (QIAGEN, USA). All DNA samples were quantified by Qubit 4.0 (Thermo Fisher Scientific, USA).

### Capture-based mtDNA sequencing

Capture-based mtDNA sequencing was performed as previously described^14^. In brief, genomic DNA was used to construct the NGS library. Then, the NGS libraries were hybridized with homemade biotinylated mtDNA capture probes. Finally, the captured mtDNA libraries were sequenced for 150 bp paired-end reads on the HiSeq XTen (Illumina) platform.

### Somatic mtDNA mutation calling and copy number calculation

Sequencing data processing and mtDNA mutation calling were carried out as described in our previous study^15^, in which the accuracy of the present method in detecting mutations at a heteroplasmy level above 1% was validated. The heteroplasmy level of each variant was determined by variant allele frequency (VAF), which was calculated as the percentage of mutant reads in the total reads for a given mutation site. A series of filter criteria were applied to accurately call mtDNA mutations, including (i) at least three reads in each strand supporting the alternative allele; (ii) total sequencing coverage ≥100×; and (iii) variant allele frequency (VAF) ≥ 1% on both strands. The tumor somatic mutations were defined as variants with VAF ≥ 1% in tumor tissues and VAF < 0.5% in paired PBMC and adjacent nontumor tissue samples. In addition, stringent inclusion criteria were applied for C > A mutations (VAF ≥ 10%) to avoid oxidative 8-oxoG-mediated errors during sample preparation. The relative mtDNA copy number was calculated as previously described^16^, in which six nDNA probes from different chromosomal locations were combined as the nDNA reference using the following formula: $\frac{mtDNA\,average\,seqencing\,depth}{average\,sequencing\,depth\,of\,reference\,gene}\times 2$

### Whole-exome sequencing (WES) and nDNA mutation calling

A paired-end WES DNA library was constructed according to the manufacturer’s instructions (Agilent). For the bilateral OC tumor tissues (*n* = 4) and matched adjacent nontumor tissues (*n* = 2) from patients 31 and 21, construction of the WES library was performed using Agilent SureSelect Human All Exon V6 (Agilent). Finally, the constructed WES libraries were sequenced for 100 bp paired-end reads on the DNBseq (BGI) platform. Quality control, mapping variant calling and annotation were carried out as previously described^5^. Somatic mutations of >5% were determined by comparing tumor and paired nontumor tissues for clonal origin analysis.

### Determination of clonality index (CI) and cutoff value

The clonal relatedness of bilateral ovarian tumors was determined by CI, which was calculated as previously described^17^ using the following formula: $CI=-lo{g}_{10}{\prod }_{m=1}^{M}P{(X)}_{m}$. M was defined as all the shared mutation sites of SBOC, and m was defined as each of the shared mutation sites. To define the cutoff value for OC clonal relatedness, the mtDNA mutation data of 103 OC patients from The Cancer Mitochondria Atlas (TCMA) were used for the generation of a positive control dataset (clonally related) and a negative control dataset (clonally unrelated). For the positive control dataset, 40%, 60% and 80% of the set of mutations from the 103 OC patients were duplicated to simulate the heterogeneity between biologically related bilateral tumor samples. For the negative control dataset, the mutations were randomly mixed from an equivalent number of pairs (3 × 103 = 309) of clonally unrelated OC patients in TCMA. After calculating the CI of all patients, the ROCR R package was used (https://ipa-tys.github.io/ROCR/) to calculate the cutoff value, which was determined to be 4.07 with a sensitivity of 0.919 and specificity of 0.984. For two OC patients, including one patient denoted as monoclonal and one patient denoted as multiclonal origin based on mtDNA-based analysis, whole-exome sequencing (WES) of the nuclear genome was further utilized to validate their clonal origin as previously described^18^.

### Phylogenetic tree construction

The phylogenetic tree of mtDNA somatic mutations was generated by DARwin6 v6.0.021 (http://darwin.cirad.fr/) for each patient to assess the metastatic pattern. All somatic mutations were converted to continuous data based on heteroplasmy level. Then, a data matrix of somatic mutation and heteroplasmy level with sample names in rows (X) and mutation site in columns (Y) was generated. The Euclidean metric between X and Y was calculated to obtain the Euclidean distance for phylogenetic tree construction.

### Calculation of mtDNA-based tumor evolutionary score (MTES)

MTES, defined as the average value of pairwise correlation coefficients across multiple sampling spots of the same patient, was used to quantify the evolutionary pattern of each patient. The data matrix of somatic mutations and heteroplasmy level was generated for correlation analysis. Pearson correlation was used to determine the correlation of the paired samples from each patient. All analyses were performed using Pandas Correlation version 1.4 (https://pandas.pydata.org).

### Statistical analysis

Statistical analysis was performed by SPSS 26.0 software. The Mann‒Whitney *U* test was used for comparisons of continuous variables between two groups, and one-way analysis of variance (ANOVA) was used to test differences among more than two groups. For paired samples, comparisons were made using paired Student’s t test. Pearson correlation analysis was used to test correlations between measured variables. All statistical analyses were two-sided, and *P* values of less than 0.05 were considered statistically significant.

## Results

### Profiling of mtDNA somatic mutation and copy number variation in ovarian cancer tissues

**Fig. 1:**
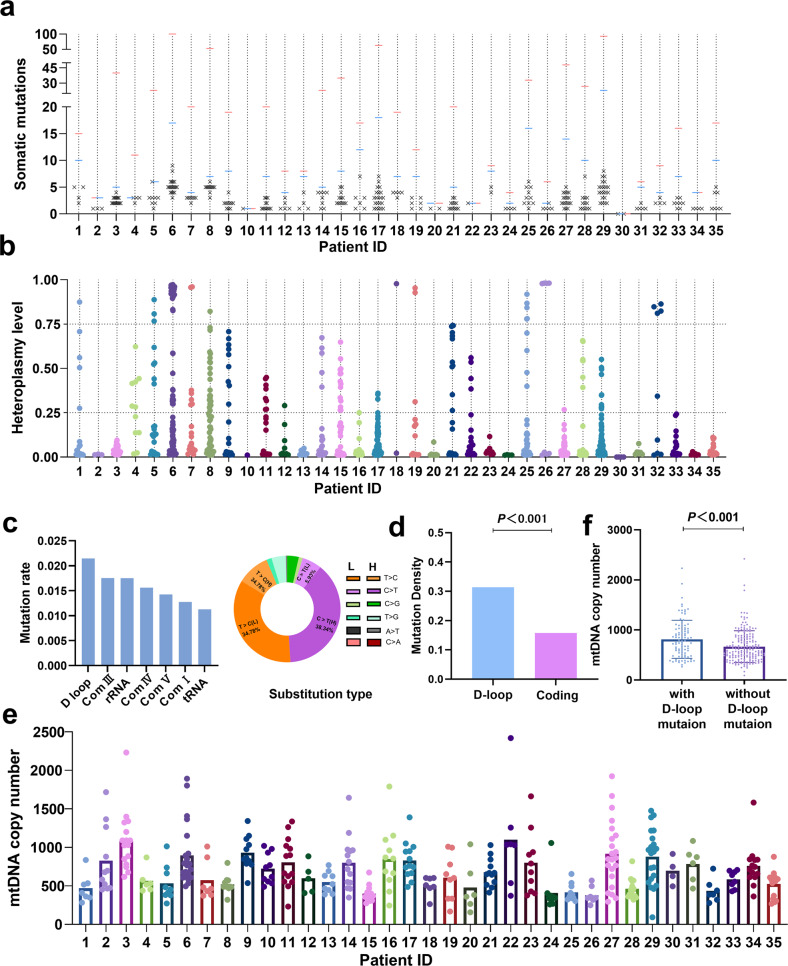
Profiling of mtDNA somatic mutation and copy number variation in ovarian cancer tissues. **a** Number of detected somatic mtDNA mutations in each sample of a patient (labeled as “x”), number of mutation sites per patient (blue dash) and total number of mutations per patient (red dash). **b** Heteroplasmy level of all somatic mutations detected in each patient. **c** Histogram of the somatic mtDNA mutation rate across the mitochondrial genome (left panel) and pie diagram showing the base substitution distribution of mtDNA mutations. Mutations are denoted based on pyrimidine base. H, H-strand; L, L-strand. **d** Comparison of mutation density between the mitochondrial D-loop and coding regions. Mutation density was defined as somatic mutation number per kilobase (kb) in one sample. **e** Distribution of mtDNA copy number for different samples of each patient. **f** Comparison of mtDNA copy number between samples with and without mtDNA D-loop mutation.

To explore the evolutionary patterns, mtDNA was sequenced in 195 primary and 200 metastatic tumor tissues from 35 OC patients. A total of 253 somatic mtDNA mutations were detected in 277 of 395 (70.13%) tumor tissues, with a range of 1–9 mutations per sample and 2-23 different mutations per patient (Fig. 1a). The mtDNA heteroplasmy level of each mutation is shown in Fig. 1b, with remarkable variation in each patient. The region-specific mtDNA mutation rate in the mitochondrial genome is shown in Fig. 1c. Mutation spectrum analysis showed that T > C (113/253, 44.66%) and C > T (112/253, 44.27%) transitions were the most frequent mutation types (Fig. 1c). Further analysis of these 253 mtDNA mutations identified significant transition dominance (225/253, 88.93%) and significant strand asymmetry (Supplementary Fig. 1a), with H-strand C > T accounting for 86.61% (97/112) and L-strand T > C accounting for 77.88% (88/113), consistent with a mitochondria-specific mutation signature^19^. Moreover, our data showed that the mutation density of the mtDNA D-loop region was significantly higher than that of the mtDNA coding region (Fig. 1d). Considering that the D-loop region is essential for mtDNA replication, we investigated the relationship between mtDNA copy number and mutations in the D-loop region. The mtDNA copy number of the samples from each patient is shown in Fig. 1e. As shown in Fig. 1f, the mtDNA copy number of tissue samples with D-loop mutations was significantly higher than that of samples without D-loop mutations.

### Distinct mtDNA mutational pattern between primary and metastatic OC tissues

**Fig. 2:**
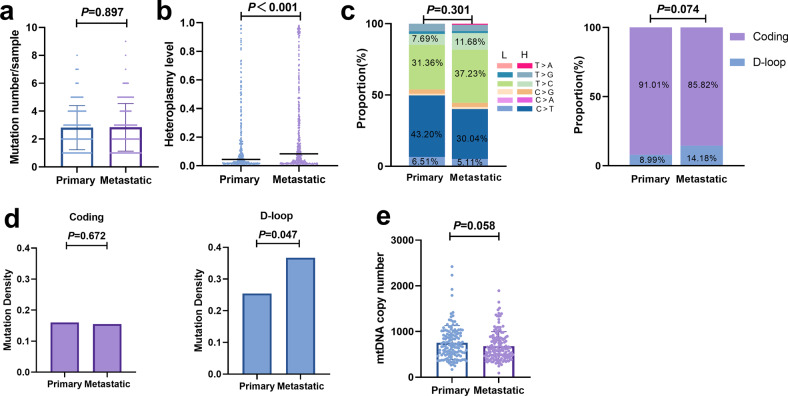
Comparison of mtDNA mutation profiles between primary and metastatic OC tissues. All comparisons were between primary and metastatic tumor tissues unless otherwise specified. **a** Somatic mutation numbers per sample. **b** Heteroplasmy levels. Bar represents median values. **c** Base substitution types and mutation proportions in mtDNA D-loop and control regions. Mutations are denoted based on pyrimidine base. H, H-strand; L, L-strand. **d** Mutation density in mtDNA D-loop and coding regions between primary and metastatic OC tissues. **e** mtDNA copy number.

We further examined the difference in mtDNA mutations in primary and metastatic tumor tissues. As shown in Fig. 2a, no significant difference in mutation numbers per sample was observed between primary and metastatic tumor tissues. However, the heteroplasmy level of metastatic tumors was significantly higher than that of primary tumors (*P* < 0.001) (Fig. 2b). As shown in Supplementary Fig. 1b, a two-way shift (increase or decrease) in the heteroplasmy level was commonly observed between primary and metastatic samples, suggesting the potential evolutionary selection of mtDNA mutations in the metastatic process. Moreover, no significant difference in base substitution types or proportion of mutations in D-loop and coding regions were observed between primary and metastatic tumor tissues (Fig. 2c). Notably, although the higher proportion of C > T (49.71%) than T > C (39.05%) mutations in primary OC tissues was consistent with the general distribution of somatic mtDNA mutations in cancer^19,20^, we observed a higher proportion of T > C (48.91%) than C > T (35.15%) mutations in metastatic OC tissues (Fig. 2c). Our results also showed that the mutation density of the D-loop region in metastatic tumor tissues was significantly higher than that in primary tumor tissues, while no remarkable difference was observed in the coding region (Fig. 2d). Furthermore, no significant difference in mtDNA copy number was found between primary and metastatic tumor tissues (Fig. 2e). These results suggest that evolutionary processes may be involved in mutational shaping during OC metastasis.

### Different mutational spectra between shared and private mutations in primary and metastatic tumor tissues

**Fig. 3:**
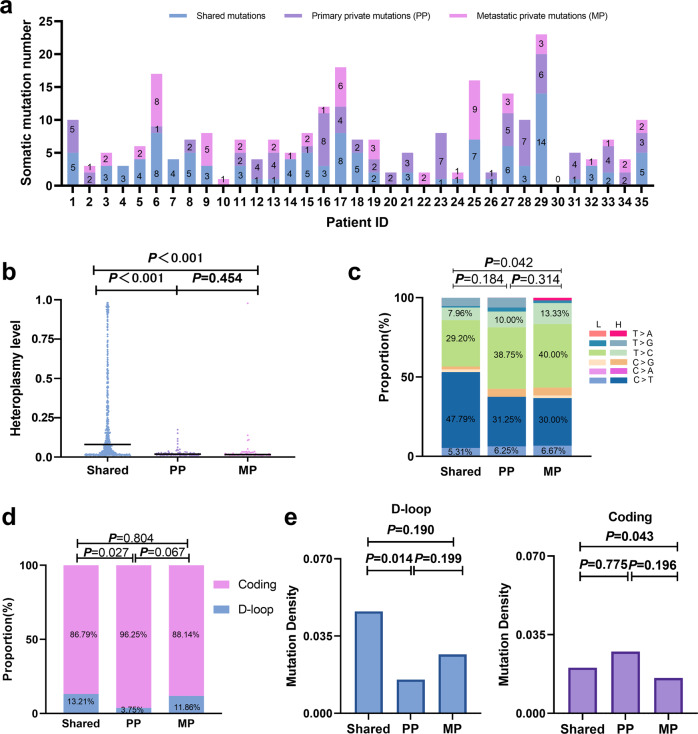
Different mutational spectra between shared and private mutations. **a** Number of shared mutations (blue), primary private mutations (PP, purple), and metastatic private mutations (MP, pink) in each patient. **b** Heteroplasmy levels among the three groups. Bar represents median values. **c** Base substitution spectrum among the three groups. Mutations are denoted based on pyrimidine base. H, H-strand; L, L-strand. **d** Mutation proportions in mtDNA D-loop and coding regions among the three groups. **e** Mutation density in mtDNA D-loop and coding regions among the three groups.

A total of 113 (44.66%) shared mtDNA mutations (defined as mutations detected in more than two tumor tissues) were identified, while 80 (31.62%) primary private (PP, private mutations in primary OC tissues) and 60 (23.72%) metastatic private (MP, private mutations in metastatic OC tissues) mutations were identified. The number of shared and private mutations in each patient is shown in Fig. 3a, revealing considerable intratumor heterogeneity. Notably, our data showed that the heteroplasmy level of shared mutations was significantly higher than that of primary private and metastatic private mutations (Fig. 3b), indicating that the shared mutations were more likely to be transmitted during evolutionary processes. Interestingly, unlike the shared mutations, both primary private and metastatic private mutations featured a higher proportion of T > C than C > T mutations, with persistent transition dominance and strand asymmetry supporting them as genuine mtDNA mutations (Fig. 3c). Moreover, the primary private mutations showed a significantly decreased proportion of D-loop mutations compared to the shared mutations (*P* < 0.05) (Fig. 3d). Further analysis confirmed that the primary private mutations in the D-loop region exhibited significantly lower density than shared mutations (*P* < 0.05) (Fig. 3e). These observations suggest that the mutagenesis of private mutations may be different from that of shared mutations in OC.

### Delineating the clonal origin of bilateral ovarian cancer tissues

**Fig. 4:**
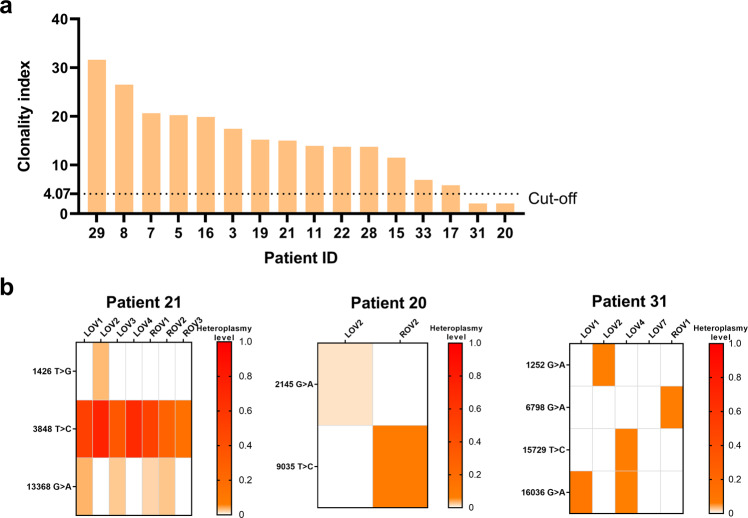
Delineation of the clonal origin and evolutionary trajectory in bilateral ovarian cancer tissues. **a** Clonality index for the 17 cases of bilateral ovarian cancer (BOC). The clonality index was defined as the likelihood of two carcinomas sharing mutations not expected to have co-occurred by chance. Black dotted lines indicate the cutoff value to define clonal relatedness. **b** Representative mtDNA mutation heatmap of monoclonal (patient 21) and polyclonal (patient 26 and 31) BOC patients showing the respective mutation sites and heteroplasmy levels. The level of heteroplasmy was color coded. LOV left ovary tumor samples, ROV right ovary tumor samples.

Because OC often occurs bilaterally, it is critical to determine the origins and relatedness of bilateral ovarian cancer (BOC). Based on the clonal theory of tumorigenesis, the monoclonal origin of SBOCs is assumed to inherit a similar set of somatic mtDNA mutations. Thus, the mtDNA mutation profiles in 16 BOC patients were analyzed, and the clonality index (CI) was further calculated. Based on a CI cutoff value of 4.07, a monoclonal origin was identified in 14 BOC patients, whereas a multiclonal origin was identified in the other 2 BOC patients (Fig. 4a). The mutation heatmap of one representative case with monoclonal origin (patient 21) and two cases with multiclonal origin (patients 20 and 31) is depicted in Fig. 4b, showing that the 3848 T > C mutation of patient 21 was shared between bilateral ovarian tumors, consistent with a monoclonal origin (left panel), whereas the 9035 T > C mutation of patient 20 was private in the right ovarian tumor and the 6798 G > A mutation of patient 31 was private in the right ovarian tumor, in line with a multiclonal origin (right panel). Furthermore, whole-exome analysis of the nuclear genome confirmed the clonal origins for these two OC patients (Supplementary Fig. 2).

### Distinct metastatic patterns of OC cells traced by mtDNA mutations

**Fig. 5:**
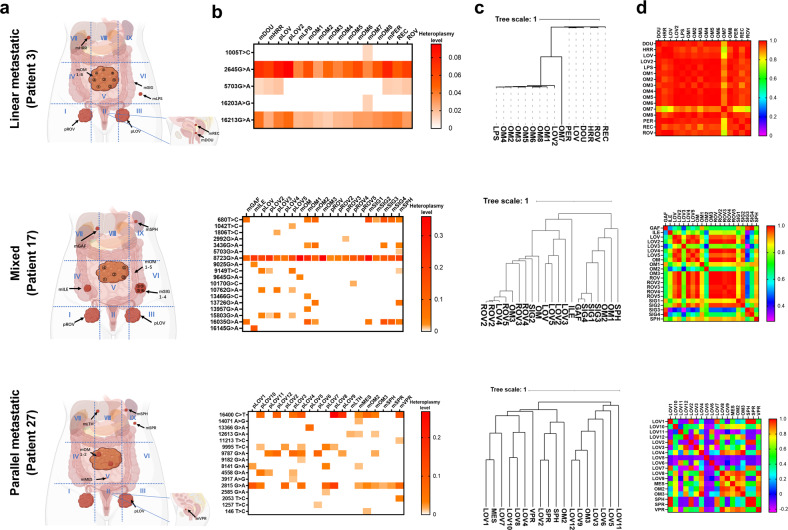
Distinct metastatic patterns of ovarian cancer traced by mtDNA mutation. Three representative cases (patient 3, patient 17, and patient 27) with distinct metastatic patterns (linear, mixed, and parallel) are shown. **a** Diagram of the spatial locations of samples in three patients. The abdominal cavity was divided into nine regions with blue dashed lines and represented by Roman numerals. The arrows indicate the locations and names of samples. **b** Heatmap showing the detailed pattern of somatic mutations and their respective heteroplasmy levels in different sampling locations of the three patients. The transverse axis is the sample name, with “p” and “m” representing the primary and metastatic sites, the longitudinal axis is the somatic mutation site, and the color in the square from white to red represents the heterogeneity level from low to high. **c** Phylogenetic tree of the three representative cases based on hierarchical clustering of mtDNA mutation and heteroplasmy. The length of the line segment represents the difference between branches, and the scale of the tree is on the left side. **d** Heatmap showing the level of pairwise correlation between the samples of the patients. The correlation values represented by colors within the squares are scaled on the right.

To ascertain the metastatic patterns from primary OC to metastatic organs, we mapped the spatial phylogenies based on mtDNA somatic mutations. The multiregion sampling and heatmaps of mtDNA somatic mutations of primary and metastatic tissues are depicted in Fig. 5a, b. The phylogenic tree was constructed based on Euclidean distances of the detected mtDNA somatic mutations among all primary and metastatic tumor tissues of each case (Fig. 5c). Interestingly, by analyzing the intratumor divergence of mtDNA mutations among primary and multiple metastatic tissues, three distinct patterns of OC metastasis were inferred, namely, linear, parallel, and mixed metastasis models, which were represented in three cases (patients 3, 27 and 17). As shown in Fig. 5b, c, the linear metastasis model in patient 3 exhibited a low degree of mtDNA mutation heterogeneity and a short evolutionary distance (left panel), whereas the parallel metastasis model in patient 27 exhibited a high degree of mtDNA mutation heterogeneity and long evolutionary distances (right panel). We considered that in the linear model, metastatic competence is probably acquired more recently in the primary tumor, resulting in low genetic divergence between the metastatic and primary tumors; in the parallel model, tumor cells may acquire dissemination potential at an earlier stage, and early disseminated cells could evolve separately at distant sites, thus generating high genetic divergence between metastatic and primary tumors. In the mixed model, the metastatic pattern consists of both linear and parallel characteristics; hence, the genetic divergence between metastatic and primary tumors is medium. Consistently, the heatmap of Pearson correlation coefficients, which was calculated by pairwise correlation of mtDNA mutations for each sampling location of primary and metastatic OC tissues, showed high correlation suggestive of high intratumor similarity in the linear metastasis model and low correlation suggestive of high intratumor divergence in the parallel metastasis model (Fig. 5d). In a fraction of OC cases, a mixed metastasis model was observed (Fig. 5b–d, middle panel). Additional representative cases supporting linear and parallel metastasis models are shown in Supplementary Fig. 3.

### Association of different metastatic patterns with treatment efficacy

**Fig. 6:**
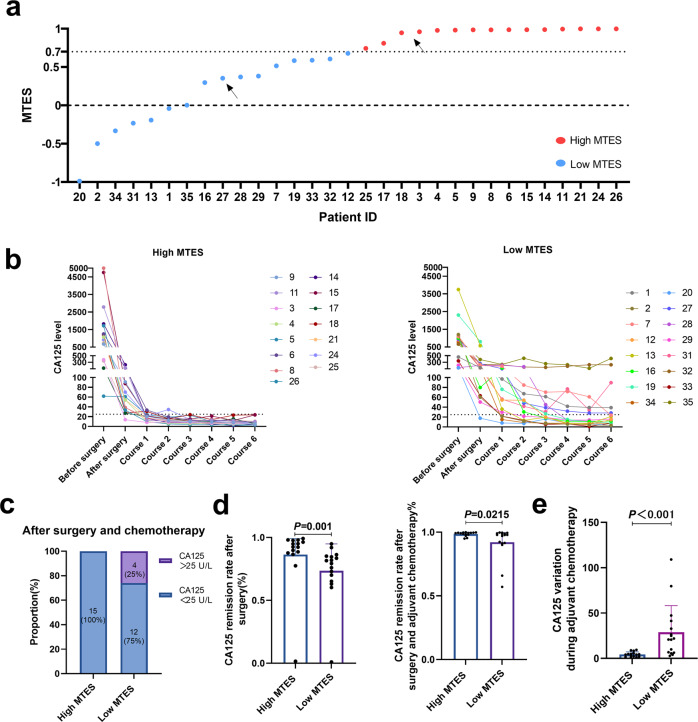
Association of mtDNA-based tumor evolutionary score (MTES) with therapeutic efficacy in ovarian cancer. **a** MTES for all patients, sorted in ascending order (from low to high). The patients were divided into high MTES (red) and low MTES (blue) groups based on the median value. The arrow indicates the representative cases shown in Fig. 5 (patient 3 and patient 27). **b** Time course change in CA125 values in the two groups before and after surgery and during six courses of adjuvant chemotherapy. The normal CA125 value is less than 22.5 U/L, marked by dashed lines. **c** Proportion of patients with normal CA125 values in the two groups after surgery and chemotherapy treatment. **d** CA125 remission rate after surgery (left panel) and after surgery and adjuvant chemotherapy (right panel) in the two groups. The CA125 remission rate was calculated as the ratio of the decreased CA125 level after treatment to the CA125 level before treatment. **e** Comparison of CA125 variation during six courses of adjuvant chemotherapy in the two groups. The variation was measured by the standard deviation (SD) of the CA125 level during chemotherapy.

The mtDNA-based tumor evolutionary score (MTES) was calculated based on the average Pearson correlation coefficient of mutations among primary and metastatic tumor tissues. The OC patients were arbitrarily divided into two groups based on the median MTES, with the high MTES group enriched with linear evolution cases and the low-MTES group enriched with parallel evolution cases (Fig. 6a). A comparison of mtDNA mutational characteristics between the high and low MTES groups is shown in Supplementary Fig. 4. In addition, the clinical relevance of MTES was explored (Fig. 6b–e). The CA125 level, recognized as a biomarker of OC therapeutic efficacy, was used to evaluate the therapeutic response in OC patients with different MTESs. Our data showed that all patients (15/15, 100%) in the high MTES group exhibited normal levels of CA125 after receiving OC treatment consisting of debulking surgery and six courses of chemotherapy (Fig. 6c, left panel). However, 25% of patients (4/16) in the low MTES group still had abnormal levels of CA125 after receiving OC treatment (Fig. 6c, right panel). Furthermore, a significant difference in the CA125 remission rate was observed between the high and low MTES groups. Our results showed that the high MTES group had higher CA125 remission than the low MTES group after surgery and chemotherapy (Fig. 6d). In addition, the OC patients in the low MTES group had higher fluctuations in CA125 levels, indicated by the variation in CA125 levels during chemotherapy, than those in the high MTEs group (Fig. 6e). Taken together, our findings suggest that OC patients with distinct metastatic patterns may respond differently to OC treatments, and patients with parallel metastatic patterns may be more resistant to standard treatments.

### Tracing tumor-derived mutations in plasma and ascitic fluid samples

**Fig. 7:**
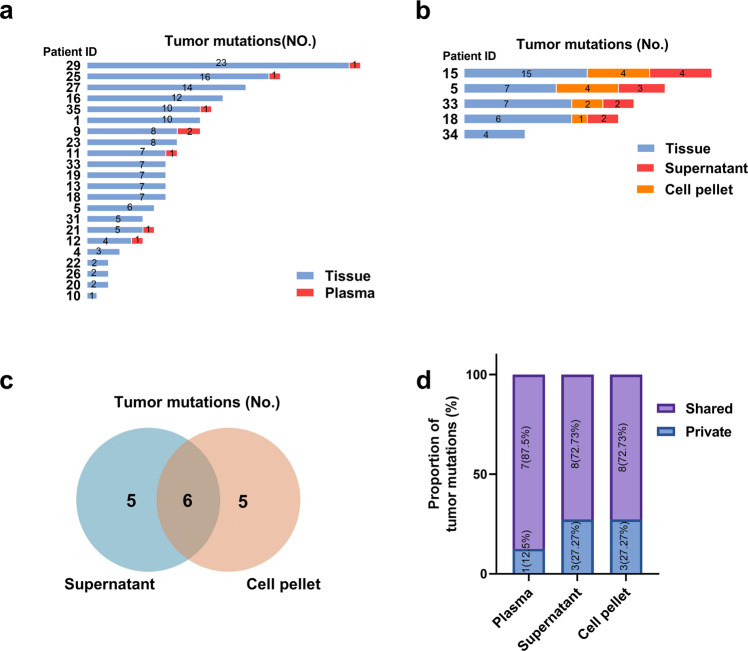
Tracing tumor-derived mutations in plasma and ascitic fluid samples. **a** Bar plot of tumor-specific mtDNA mutations detected in plasma samples from 22 patients. Mutations detected in plasma are shown in red, and mutations observed in tumor tissues are shown in blue. **b** Bar plot of tumor-specific mtDNA mutations detected in the cell pellet fraction (orange) and supernatant fraction (red) of ascitic fluids from 5 patients. Mutations observed in corresponding tumor tissues are shown in blue. **c** Venn diagram showing the level of overlap for tumor-specific mutations between the cell pellet and supernatant fraction of ascitic fluids. **d** Proportion of shared and private mutations for tumor-specific mutations detected in plasma, ascitic supernatant, and ascitic cell pellet samples.

We also detected tumor-derived mtDNA mutations in preoperative plasma and ascitic fluid samples. As shown in Fig. 7a, tumor-specific mutations (range 3–24) were identified in OC tissue samples from all 22 patients whose plasma samples were available for DNA extraction and sequencing. These mutations were detected in paired plasma samples (range 1–2) in only 7 of 22 (31.82%) OC patients. In comparison, of the 5 OC patients with available ascitic fluid samples, the tumor-specific mutations (range 4–15) identified in the OC tissue samples were detected in ascitic fluid supernatant samples (range 2–4) and cell pellet samples (range 1–4) in 4 (80%) patients (Fig. 7b). Then, the tumor-derived mutations detected in supernatant and cell pellet samples were compared. Our data showed that 6 of 16 mutations were detected in both the supernatant and cell pellet, while 5 of 16 and 5 of 16 mutations were uniquely detected in supernatant and cell pellet samples, respectively (Fig. 7c). In addition, we further investigated whether the tumor-derived mutations in plasma and ascitic fluid samples were shared or private. Our results showed that 7 of 8 (87.5%), 8 of 11 (72.7%) and 8 of 11 (72.7%) mutations detected in plasma, ascitic supernatant and cell pellet samples, respectively, were shared mutations (Fig. 7d). Collectively, these results indicate that tumor-derived mutations are more likely to be detected in ascitic fluid than in plasma samples, consistent with the theory that OC metastasis occurs more frequently by invading the abdominal cavity than by spreading through the blood system.

## Discussion

Compared with nuclear DNA, mtDNA is unique in its small size, high copy number, and high mutation rate, which creates several advantages suitable for lineage tracing: the high mutation rate provides a substantial target for detecting mtDNA genetic diversity; the small size allows for low sequencing cost and an easy analysis pipeline; and the high copy number enables the evaluation of mtDNA copy number change at different temporal/spatial sites, which commonly represents the mitochondrial biogenesis process. In this study, by multiregional high-depth mtDNA sequencing and analysis of 35 advanced-stage OC patients, we comprehensively characterized OC heterogeneity, clonality, metastatic patterns, and their clinical relevance. Our data support several key findings. First, the mtDNA mutational patterns of primary and metastatic tumors were distinct. Second, most bilateral ovarian tumors in our cohort were monoclonal based on mtDNA mutation analysis. Third, the distinct metastatic patterns of OC patients traced by mtDNA mutations were associated with different clinical treatment efficacies. These findings provide novel insights into the evolution and metastatic trajectories of OC patients and facilitate novel strategies for precision therapy. Previous pancancer analysis of mtDNA mutations has revealed a distinct mtDNA mutation signature featuring transition dominance, strand asymmetry, and a higher proportion of C > T than T > C mutations (H-strand and L-strand together)^19,20^. In our study, very interestingly, a higher proportion of C > T than T > C was observed only in primary OC tissues. By classifying the mtDNA mutations into shared, primary private and metastatic private, we further showed that the opposite pattern, a higher proportion of T > C than C > T mutations, was a feature of private mtDNA mutations (primary private and metastatic private) in OC. Notably, our study is unique in its use of multispot sampling, thus allowing the differentiation of shared and private mtDNA mutations. Recently, Mikhailova et al. reported that increased T > C mutation is a specific age-associated mtDNA mutation signature^21^. The potential relationship between these two processes needs to be elucidated in future studies.

Given that advanced-stage OC commonly affects both ovaries, interrogating the monoclonal or polyclonal origin of bilateral ovarian cancers (BOCs) is critical for deciding therapeutic options^22^. Several previous studies have attempted to ascertain the clonal origins of bilateral ovarian tumors using various experimental strategies^18,23,24^. Analysis of X-chromosome inactivation and microsatellite instability (MSI) has suggested that bilateral ovarian tumors share a unifocal origin^23^. By whole-genome sequencing of 12 BOC patients, a recent study also reported the monoclonality of BOC^18^. Additionally, based on loss of heterozygosity (LOH) analysis, Abeln et al. identified 14 of 16 BOC cases supporting a monoclonal model, while the other two cases suggested polyclonal origins^24^. In the present study, we applied systematic mtDNA mutational profiling of BOCs for the first time (to our knowledge) and revealed a monoclonal origin in 14 of 16 BOC patients and a polyclonal origin in 2 of 16 BOC patients using a VAF threshold of ≥1%. Future efforts for precision detection of mtDNA mutations with a lower VAF may greatly increase the number of mutation sites for more accurate clonality analysis. Our data suggest that the mtDNA sequencing-based approach may be incorporated into clinical histopathology to accurately classify the clonal origins of bilateral ovarian tumors.

Ovarian cancers often metastasize widely within the peritoneal cavity, making it challenging to delineate distinct metastatic patterns^25^. Previous efforts to understand OC metastasis have relied mostly on genetic markers in the nuclear genome^26–28^. For instance, Masoodi et al. analyzed nuclear mutations in a small sample of six OC patients. Among them, four patients showed substantial sharing of somatic mutations between primary and metastatic tumors, consistent with the linear metastasis model, whereas two patients exhibited extensive mutation diversification between primary and metastatic tumors, consistent with the parallel metastasis model^5^. Given growing evidence that somatic mtDNA mutations are efficient lineage-tracing markers in cancer^9,29^, we applied high-depth mtDNA sequencing to delineate metastatic patterns in 35 OC patients and successfully classified OC cases into linear and parallel metastasis modes based on the bimodal distribution of mtDNA mutation heterogeneity and evolutionary distance. Our results indicate that OC cancer with a parallel metastasis mode exhibits a high degree of intratumor heterogeneity and evolutionary divergence. Due to the limited discriminative power of mtDNA bulk sequencing, our current mtDNA-based lineage tracing can be further improved by mtDNA single-cell sequencing, which may allow more accurate dissection of the clonal composition for each tumor tissue and more accurate delineation of mutational and evolutionary timing for different tumor clones in OC.

A personalized therapeutic strategy based on distinct metastatic patterns is urgently needed. Previous studies have reported that PTEN mutations in breast cancer or EGFR mutations in non-small cell lung cancer metastatic tumors influence the metastatic pattern and further increase resistance to therapy^30,31^. In the present study, we classified OC patients into distinct metastasis modes based on the mtDNA-based tumor evolutionary score (MTES), which provided an overall measure of tumor similarity across multiple sampling locations at the mtDNA mutation level. We found that OC patients with distinct metastasis modes responded differently to OC treatments. Our results also suggest that patients with the parallel metastasis mode (low MTES) may be more resistant to standard treatments, for whom more courses of adjuvant chemotherapy are needed. It is conceivable that patients with low MTES may experience more early-stage cancer dissemination and develop higher clone heterogeneity, which could potentially contribute to their unfavorable therapeutic response. As proof of concept, tumor-specific mutations in OC tissues can more easily be identified in ascitic fluid samples than in plasma samples, supporting that ascitic fluid may be more suitable to serve as a potential clinical laboratory analyte for OC patients.

In the present study, we aimed to make full use of mtDNA-based analysis advantages, such as high copy number and mutation rate, low sequencing cost and easy analysis pipeline. Therefore, mtDNA-based analysis could provide certain novel insights that cannot be captured by gDNA-based analysis. For instance, the unique multicopy feature of mtDNA enables the evaluation of mtDNA copy number changes at different temporal/spatial sites, which commonly represents the mitochondrial biogenesis process. In addition, a series of previous studies have reported that certain mtDNA mutations could potentially alter the metabolic function or redox homeostasis of mitochondria, which may affect the metastasis and prognosis of cancer patients^9–11^. Thus, mtDNA-based analysis may help identify functional mutations, especially those associated with mitochondrial metabolism and metastasis. In summary, the present study presents a comprehensive delineation of OC metastatic patterns by tracing mtDNA mutations via multiregion sampling of primary and metastatic tumors, which holds great promise for improving the treatment of OC patients. Despite the inspiring findings, the present study is based on high-depth mtDNA sequencing at the bulk level, which makes it challenging to determine whether the identified private mtDNA mutations were newly developed during OC metastasis or preexisting at levels below the limit of detection in the primary tumor. Future studies using mtDNA single-cell sequencing may resolve clonal evolution and metastasis at a higher resolution, providing further clinical insight into OC metastasis.

## Supplementary information

Supplementary Materials

## Data Availability

The mtDNA variant and nDNA somatic mutation data generated in the present study are available in the National Genomics Data Center Genome Variation Map database (https://ngdc.cncb.ac.cn/gvm/) under accession number PRJCA013770. Public data on mtDNA mutations from 103 OC patients were downloaded from the Cancer Mitochondria Atlas (https://ibl.mdanderson.org/tcma).
