# Supplementary material for: Metastatic pattern of ovarian cancer delineated by tracing the evolution of mitochondrial DNA mutations
Source: Exp Mol Med. 2023 Jul 3;55(7):1388–98. doi: 10.1038/s12276-023-01011-2 (PMC10393968; doi:10.1038/s12276-023-01011-2)
Supplement: Supplementary file 1 — Supplementary Materials [file 12276_2023_1011_MOESM1_ESM.pdf]

Metastatic pattern of ovarian cancer delineated by tracing the evolution of mitochondrial DNA mutations

Zhiyang Xu[1#], Kaixiang Zhou[2#], Zhenni Wang[2#], Yang Liu[2], Xingguo Wang[1], Tian Gao[1], Fanfan Xie[2], Qing Yuan[2], Xiwen Gu[3], Shujuan Liu[1*], Jinliang Xing[2*]

[1]Department of Obstetrics and Gynecology, Xijing Hospital, Fourth Military Medical University, Xi'an, China.

[2]State Key Laboratory of Cancer Biology and Department of Physiology and Pathophysiology, Fourth Military Medical University, Xi'an, China.

[3]State Key Laboratory of Cancer Biology and Department of Pathology, Xijing Hospital and School of Basic Medicine, Fourth Military Medical University, Xi'an, China.

Contents

Supplementary figure 1. Mutational spectrum with 96 contexts and heteroplasmy changes of shared mtDNA mutations between primary and metastasis 2

Supplementary figure 2. Clonality analysis of bilateral ovary cancer from patient #31 and #21 based on whole exome sequencing. 3

Supplementary figure 3 Distinct metastatic patterns of ovarian cancer traced by mtDNA mutation. 4

Supplementary figure 4 Comparison of mutation characteristics between high and low MTES groups... 5

Supplementary table 1. Sampling sites and abbreviation 6

Supplementary figures

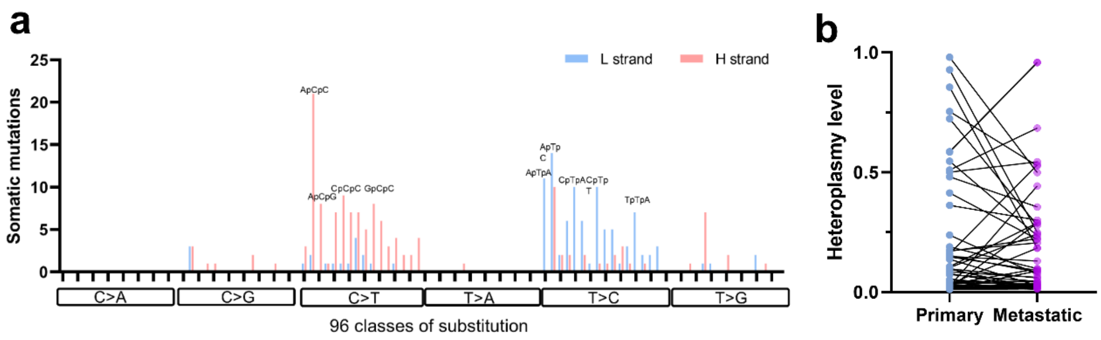

Supplementary Fig. 1. Mutational spectrum with 96 contexts and heteroplasmy changes of shared mtDNA mutations between primary and metastasis. (a) Overall spectrum of somatic mtDNA mutations identified in both primary and metastatic OC tissues from 35 patients. The mutations are denoted by pyrimidine bases according to 96 tri-nucleotide contexts, with H-strand (red) and L-strand (blue) colored differently. (b) Paired comparison of shared mtDNA mutations between primary and metastasis samples.

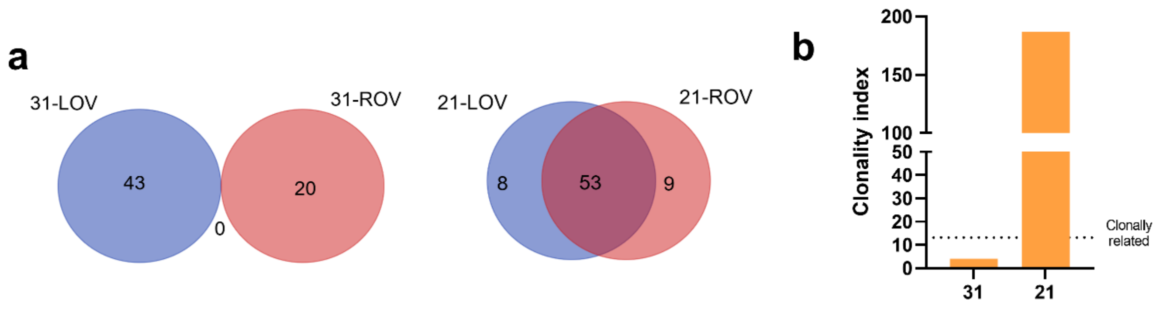

Supplementary Fig. 2. Clonality analysis of bilateral ovary cancer from patient #31 and #21 based on whole exome sequencing. (a) Pie chart showing the distribution of somatic nDNA mutations (cutoff >5%) in bilateral tumor tissues, with lack of shared mutations in patient #31 supporting independent origin and substantial shared mutations in patient #21 supporting single clonal origin. LOV, left ovary; ROV, right ovary. (b) Clonality index of the bilateral tumors in patient #31 and patient #21 based on somatic nDNA mutations.

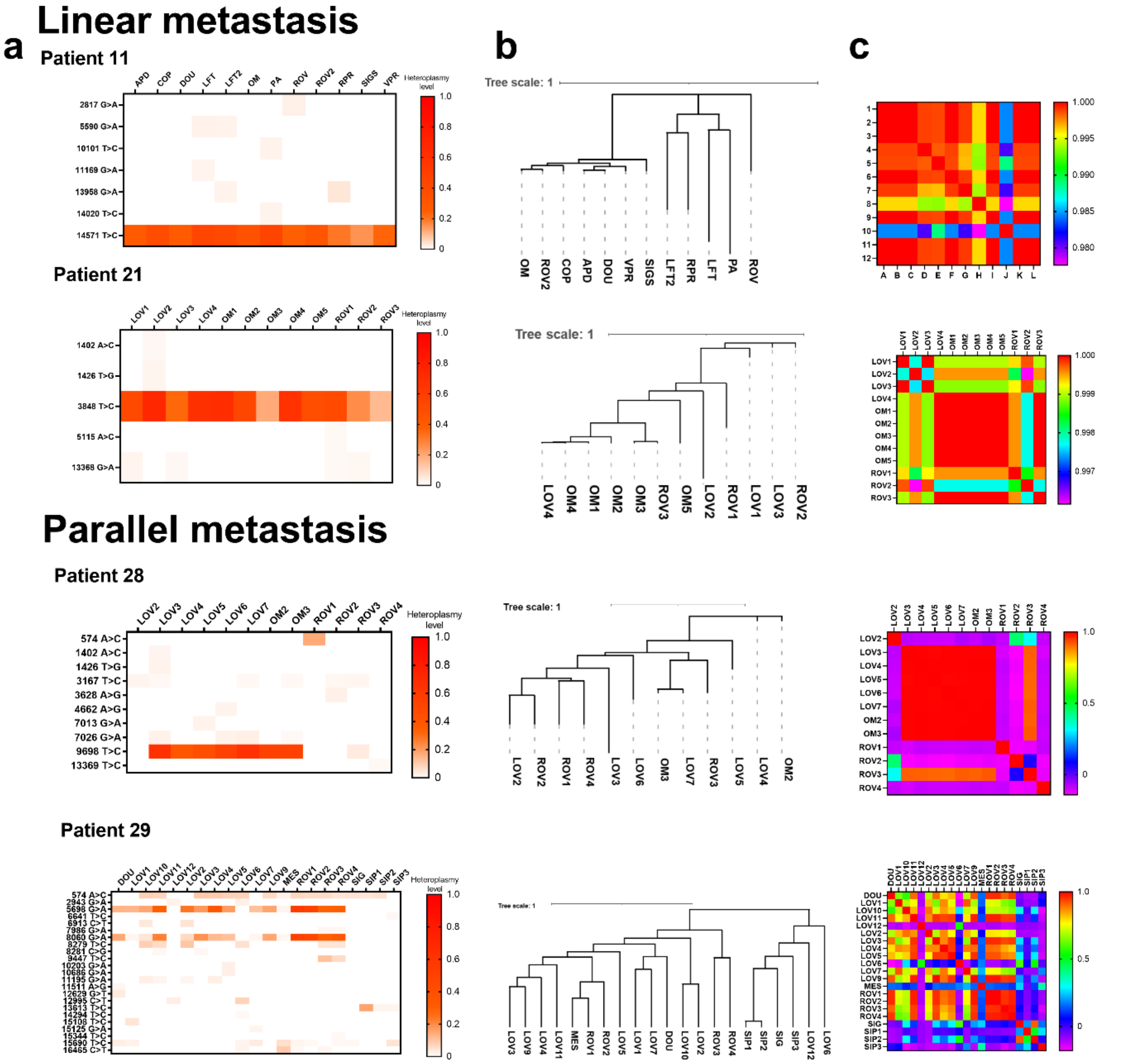

Supplementary Fig. 3. Distinct metastatic patterns of ovarian cancer traced by mtDNA mutation. Additional cases support linear and parallel evolution pattern were provided. (a) Heat map showing the detailed pattern of somatic mutations and their respective heteroplasmy level in different sampling locations of each patient. The longitudinal axis was the somatic mutation site, and the color in the square from white to red represented the heterogeneity level from low to high. (b) Phylogenetic tree of each case based on hierarchical clustering of mtDNA mutation and heteroplasmy. The length of the line segment represented the difference between branch, scale of the tree was in the left side. (c) Heat map showing the level of pairwise correlation between the samples of the patients. The correlation values represented by colors within the squares were scaled on the right.

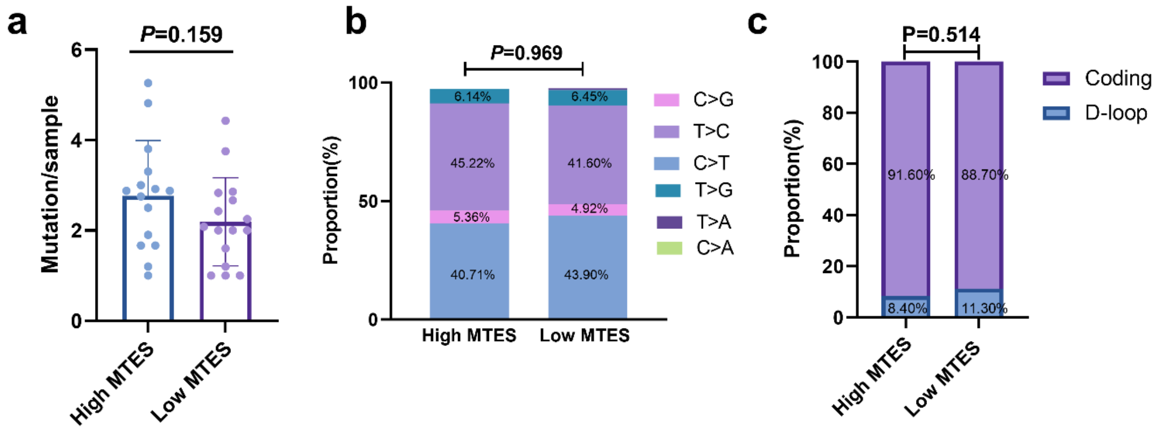

Supplementary Fig. 4. Comparison of mutation characteristics between high and low MTES groups. All the comparisons were between high and low MTES groups unless otherwise specified. (a) Mutation number per sample. (b) Base substitution types. (c) Mutation proportions in mtDNA D-loop and control regions.

Supplementary tables

Supplementary Table 1. Sampling sites and abbreviation

Zone	Samping site	Abbreviation	Zone	Samping site	Abbreviation
IV	Appendix	APD	V	Omentum	OM
VI	Colon parenchyma	COP	III	Ovarian	OV
IX	Diaphragm	DIA	X	Paraortic lymph node	PA
II	Douglas pouch	DOU	VIII	Pancreas	PAN
VII	Gallbladder	GAB	V	Peritoneum	PER
VII	Gallbladder fossa	GAF	IV	Right colon	RCO
VII	Hepatic and renal recess	HRR	VII	Right diaphragm	RDI
X	Left extrailiac lymph node	LEL	II	Rectal surface	REC
III	Left fallopian tube	LFT	I	Right fallopian tube	RFT
X	Intraliac lymph node	LIC	I	Right ovary	ROV
IV	Ileocecum	LIE	X	Para-abdominal aortic lymph nodes	RPA
X	Left inguinal lymph node	LIG	IV	Right peritoneum	RPR
X	Left intralyxal lymph node	LIL	IV	Right paracolonal sulcus	RPS
VII	Liver parenchyma	LIP	X	Renal vein lymph nodes	RVL
VII	Liver	LIV	VI	Sigmoid surface	SIG
VIII	Small omentum	LOM	V	Small intestine surface	SIN
III	Left ovary	LOV	IX	Splenic hilus	SPH
X	Left paraortic lymph node	LPA	IX	Spleen	SPL
III	Left peritoneum	LPR	VIII	Transverse mesocolic membrane	TCM
VI	Left paracolic sulcus	LPS	VIII	Transverse colon	TRC
VII	Ligamentum teres hepatis	LTH	II	Uterus	UTE
V	Mesentery	MES	III	Vesical peritoneal reflection	VPR

Note: The abdominal cavity was divided into nine regions and represented by Roman numerals. Roman numeral X referred to the lymph node.
